# A Variety of Practical Points

**Published:** 1888-10

**Authors:** 


### CULLINGS FROM CONTEMPORARIES.

EDITED BY T. J. BENNETT, M. D.

TYPHOID FEVER SPECIFIC.—Dr. W. F. Waugh, of Philadelphia, says sulpho-carbolate of zinc is the nearest approach to a specific for typhoid fever known.

MORPHINE HABIT.—Cramer accidently discovered in the tincture of castor, (castor 1, alcohol 5,) a remedy for breaking off the morphine habit.—*Medical Register*.

TO DETECT BLOOD IN THE URINE.—Ten c. c. of urine are acidified with a drop of acetic acid and shaken up with 3 c. c. of chloroform. The chloroform is then allowed to settle to the bottom, when it assumes a red color, Three drops of blood in 250 c. c. of urine can be detected.—A. LECHINI.

FOR BRONCHITIS.—Hydrate of terpina, (dose two to fifteen grains,) is an alkaloid of turpentine. It is a small crystal, resembling chloral. Is insoluble in water, soluble in glycerine, to which some pleasant syrup may be added as a vehicle.

Dr. J. H. Smith, of Dallas, calls attention to this drug as a most valuable remedy in the treatment of catarrhal affections of the lower air-passages.—*In Transactions of the Texas State Medical Association, 1888.*

HERRICK'S OPERATION FOR LACERATED CERVIX.—Denude the lacerated edges in the usual way and properly coaptate them. Then, instead of introducing sutures, apply a wide elastic band, shaped like the cervix, and large enough to cover the whole os and neck, leaving only a small hole for the secretion to escape. This band, being fastened snugly over the cervix, the wound heals. The advantages claimed are: The operator needs no assistants. No anesthetic. No stitches to remove. Patient does not have to keep her bed. It is just as successful.—*Exchange.*

---

EARLY OPERATION FOR PTERYGIUM.—Dr. F. W. Marlow, Professor of Ophthalmology, University of Syracuse, advises (N. Y. Med. Journal, Aug. 25,) early removal of pterygia in all cases in which the degree of acuteness of vision is below the normal.

The doctor's opinion is based upon a number of cases in which he carefully worked out the refraction before and after the opetion. He says "the obvious explanation of the change in refraction and visual acuteness is that the drag upon the corneal tissues by the pterygium produced irregular astigmatism, and that when the pterygium was removed the regular corneal curve was resumed."

---

FOR THE PAINLESS EXTRACTION OF TEETH.—The following Recipe is sent by Dr. J. M. Lewis, of Mexia, who says that it is the formula used by the "long-haired Indian doctor," whose face and voice are so familiar on the streets of most Texas towns. Dr. Lewis says, after having injected the gum or rubbed it with this mixture, the doctor assures his patient that it "won't hurt," and there is "no danger"; and very dexterously extracts the tooth, and that, really, there is very little, if any, pain. Abscess sometimes follows in the alveola, which is due, Dr. Lewis says, to using needle not properly cleaned or to too large a dose of the mixture. This is not the first instance where the "regulars" have gotten an item from the "irregulars." By the by, upon comparison, it seems, that this Recipe is identical with the one given by Dr. S. T. Lowry at the Galveston meeting of the Texas State Medical Association, and which appears in the Transactions. We do not remember where Dr. Lowry said he got the formula—whether it was original with him or not.

℞ Cocaine Mur: cryst. gr. viii.
Chloral Hydrate, gr. v.
Acid Carbolici, gtt. iii.
Aquæ destil. ℥ iii m.

S. Inject 2 to 3 drops in the gum.

